# Factors associated with postpartum family planning use in Rwanda

**DOI:** 10.1186/s40834-023-00261-9

**Published:** 2024-01-02

**Authors:** Muzungu Hirwa Sylvain, Rwema Valens

**Affiliations:** Centre of Excellence in Data Science, University of Rwanda, Kigali, Rwanda

**Keywords:** Postpartum contraceptive, Family planning, Rwanda healthcare, Women health, Maternal health

## Abstract

**Background:**

Resumption and initiation of contraceptive methods after delivery are of critical importance in ensuring the well-being of the mother and the newborn. However, evidence related with postpartum family planning in Rwanda is scanty. This study employed survival analysis techniques to study the time to resumption or initiation of contraceptive methods after childbirth in Rwandan women and the associated factors.

**Methods:**

Data were collected from the 2020 Rwanda Demographic and Health Survey (RDHS). Descriptive statistics were obtained after adjusting for study design. Initial analysis was conducted using the Kaplan-Meier method, followed by univariate and multivariable Cox Hazard proportional models to study factors associated with the initiation of contraceptive use after delivery. Cox Model assumptions were tested using scaled Schoenfeld Residuals.

**Results:**

5682 women who delivered in the five years preceding the survey were included in this research. The percentage of women who used modern contraceptives was 32%, 55%, 67%, and 79% at one month, six months, one year and two years respectively. Delivery by cesarean section, access to health insurance, and an increase in the number of children under five years of age were associated with increased chances of utilizing modern contraceptives after delivery. An increase in women’s age and in the ideal number of children and women with a history of terminated pregnancy were associated with reduced chances of using postpartum family planning. The influence of religion was highlighted, with Catholic women associated with high contraceptive use.

**Conclusion:**

There is a need to strengthen postpartum family planning in Rwanda. Policy makers and clinicians should provide additional customized interventions for women with factors associated with low use.

## Introduction

Over the past two decades, Rwanda has reduced maternal mortality by more than 70%. The country has reduced the maternal mortality ratio from 1070 in 2000 to 203 deaths per 100,000 live births by 2020. Similarly, the percentage of contraceptive users tripled over the same period [1]. It is estimated that 40% of the maternal mortality reduction in developing nations is attributed to the increased usage of family planning services [2].

Family planning also contributes to women’s empowerment and poverty reduction [3]. Family planning allows women to accommodate their children and avoid unintended pregnancies [4].

Access to postpartum contraception is critical to reducing maternal mortality. Tightly spaced pregnancies, especially those conceived within one year after childbirth, pose additional risks for the mother and child [5]. It is estimated that child mortality would reduce by 13% and 25% if all women spaced pregnancies by at least two and three years, respectively [6]. The use of postpartum family planning provides an additional advantage in resource-constrained settings, where access to regular healthcare services is challenging. With the increasing percentage of births occurring in health facilities, the postpartum period is an opportunity to provide family planning information and services [7].

The 2020 Rwanda Demographic and Health Survey revealed that 15% of all births in Rwanda occur within two years of the previous birth [1]. A study conducted in Rwanda revealed that only 11% of women received contraceptives methods before discharge after delivery [7]. The reasons for this unsatisfactory use include limited knowledge of available methods, failure to integrate family planning into antenatal and postnatal care, and myths associated with postpartum family planning [8].

A limited number of studies have analyzed postpartum family planning in Rwanda [7, 9–12]. Two studies were conducted in urban hospital hospitals with samples that were not representative. Other studies have used conventional statistical methods, such as logistic regression, which evaluates the status of contraceptive use at a particular time. In the presence of such data, the use of survival analysis models, especially cox proportional hazard model provides more advantages than logistic regression. This is due to the fact that the Cox proportional hazards models take account of the time until events occur [13]. This study introduced the concept of survival analysis to study the time of initiation or resumption of postpartum contraceptives in Rwanda.

## Methods

### Data

This study used data retrieved from the 2020 Rwanda Demographic and Health Survey, a nationally representative survey conducted by the National Institute of Statistics with its International Partners [1].

Data on childbirths, timing of contraceptive use, method type and duration of use, were retrieved from the calendar part of the survey. The data cleaning process consisted of sampling women with at least one childbirth in the five years preceding the survey. Additionally, we selected women who had used modern family planning methods after childbirth. For women in the sample with more than one delivery, the most recent delivery was considered. We extracted information on the time of childbirth, duration from childbirth to the use of contraceptives, and contraceptive method used.

#### Dependent variable

The dependent variable was the months taken to use the modern contraceptive method after delivery. Modern contraceptives included were pills, intrauterine devices, implants, female and male condom, female and male sterilization, diaphragm and emergency contraceptives methods. Time was measured in months, starting from 1 (1 indicates the month in which she received the method) to 60 months. In addition, another variable called status was created to indicate whether an event of interest (use of contraceptives) occurred.

#### Independent variables

Independent variables were selected using existing literature on factors that are associated with family planning behavior. These are factors that are known from the literature to affect contraceptive use. They include demographic information such as age, type of residence (urban or rural), and country’s regions. Socio-economic factors such as number of children, ideal number of children, number of household members, religion, marital status, education, wealth index. In addition, we included health-related factors such as history of terminated pregnancies, place of last delivery, mode of delivery, antenatal care, and access to insurance.

### Statistical analysis

This study employed survival analysis in the study of time of resumption and initiation of modern contraceptive method after delivery. Initially, Kaplan Meier analysis was applied to study the time of initiation and resumption of modern contraceptive after childbirth. Kaplan Meier was followed by Cox proportional hazards model that was implemented using the R Programming Language Package [14]. The Cox Model was used to study the effects of independent factors on the timing of contraceptive use after childbirth [15]. Initially, bivariate Cox Model analysis was conducted and significant variables in bivariate analysis were selected for multivariate analyses.

The evaluation of the model results was based on hazard ratio, 95% confidence intervals, and its and *P* values. The significance level was set at *P* < 0.05. Covariates with *P* values below the significance level and with hazard ratios that were significantly different from one were selected for multivariate analysis. In addition, the hazard proportionality assumption was tested using the scaled Schoenfeld Residuals.

## Results

### Dataset characteristics

In total, we analysed data on 5682 women who delivered during the 5 years of the survey. The average duration of contraceptive use after delivery was 9.19 months. The mean age of women in the sample was 31.4. Most women (65%) had primary education. 75% of women were living in households that are headed by male. The descriptive statistics are presented in Table 1.

**Table 1:** Descriptive statistics of study sample

Characteristic	N = 5,682^*1*^
Respondent Current Age	31 (26, 36)
Total children ever born	3.00 (2.00, 4.00)
Number of antenatal visits during pregnancy	3.00 (3.00, 4.00)
Ideal number of children	3.00 (3.00, 4.00)
Timing 1st antenatal care (Month)	3.00 (3.00, 4.00)
Number of household members	5.00 (4.00, 6.00)
Number of children 5 and under in household	1.00(1.00, 2.00)
Antenatal care provider	
Medical doctor	239 (4.2%)
No antenatal visit	129 (2.3%)
Nurse/midwife	5,314 (94%)
Last child delivered.by	
Medical doctor	1,296 (23%)
Nurse/midwife	4,043 (71%)
Other	342 (6.0%)
Place of last delivery	
Health center	3,372 (59%)
Home	286 (5.0%)
Other	82 (1.4%)
Private clinic/polyclinic	117 (2.1%)
Provincial/district hospital	1,483 (26%)
Referral hospital	341 (6.0%)
Religion	
Adventist	733 (13%)
Catholic	1,945 (34%)
Muslim	105 (1.9%)
Other	106 (1.9%)
Protestant	2,792 (49%)
Current marital status	
Married/Living with partner	4,564 (80%)
Never in union	606 (11%)
Widowed/Divorced/Separated	512 (9.0%)
Highest Level of education	
No Education	645 (11%)
Primary	3,662 (64%)
secondary	1,148 (20%)
Higher	228 (4.0%)
Wealth Category	
Poorest	1,351 (24%)
Poorer	1,120 (20%)
Middle	1,112 (20%)
Richer	1,094 (19%)
Richest	1,005 (18%)
Type of Residence	
Rural	4,682 (82%)
Urban	1,000 (18%)
Had terminated pregnancy	897 (16%)
Delivered by cesarean section	889 (16%)
Have health insurance	4,599 (81%)
Reported problem getting money for medical purpose	2,558 (45%)
Reported problem of distance to health facility	1,318 (23%)
Respondent currently working	4,311 (76%)
Region/Province	
Kigali	770 (14%)
South	1,184 (21%)
West	1,255 (22%)
North	906 (16%)
East	1,567 (28%)
Sex of the household head	
Male	4,298 (76%)
Female	1,384 (24%)

^*1*^Median (Interquartile Range); n (%)

### Results of Kaplan Meier analysis

Kaplan-Meier estimation of the survival function was conducted in R Studio using the R programming language. We plotted Kaplan Meier Curve using R Function “Survfit” of Survival Library. Initially, we plotted Kaplan–Meier curves for the entire 60 months (5 years). We reduced the analysis time to 36 months after childbirth. Table 2 presents the Kaplan–Meier table.

**Table 2:** Kaplan Meier estimation of survival table

Time (months)	Number at risk	Number of events	Survival probability	Standard error	Lower 95% CI	Upper 95% CI
1	5682	1784	0.68	0.00625	0.668	0.692
2	3788	553	0.581	0.00661	0.568	0.594
3	3159	292	0.527	0.0067	0.514	0.54
6	2381	450	0.442	0.00672	0.429	0.455
12	1545	546	0.328	0.00653	0.315	0.341
24	669	449	0.211	0.00616	0.2	0.224
36	296	105	0.171	0.00616	0.16	0.184

**Fig. 1:**
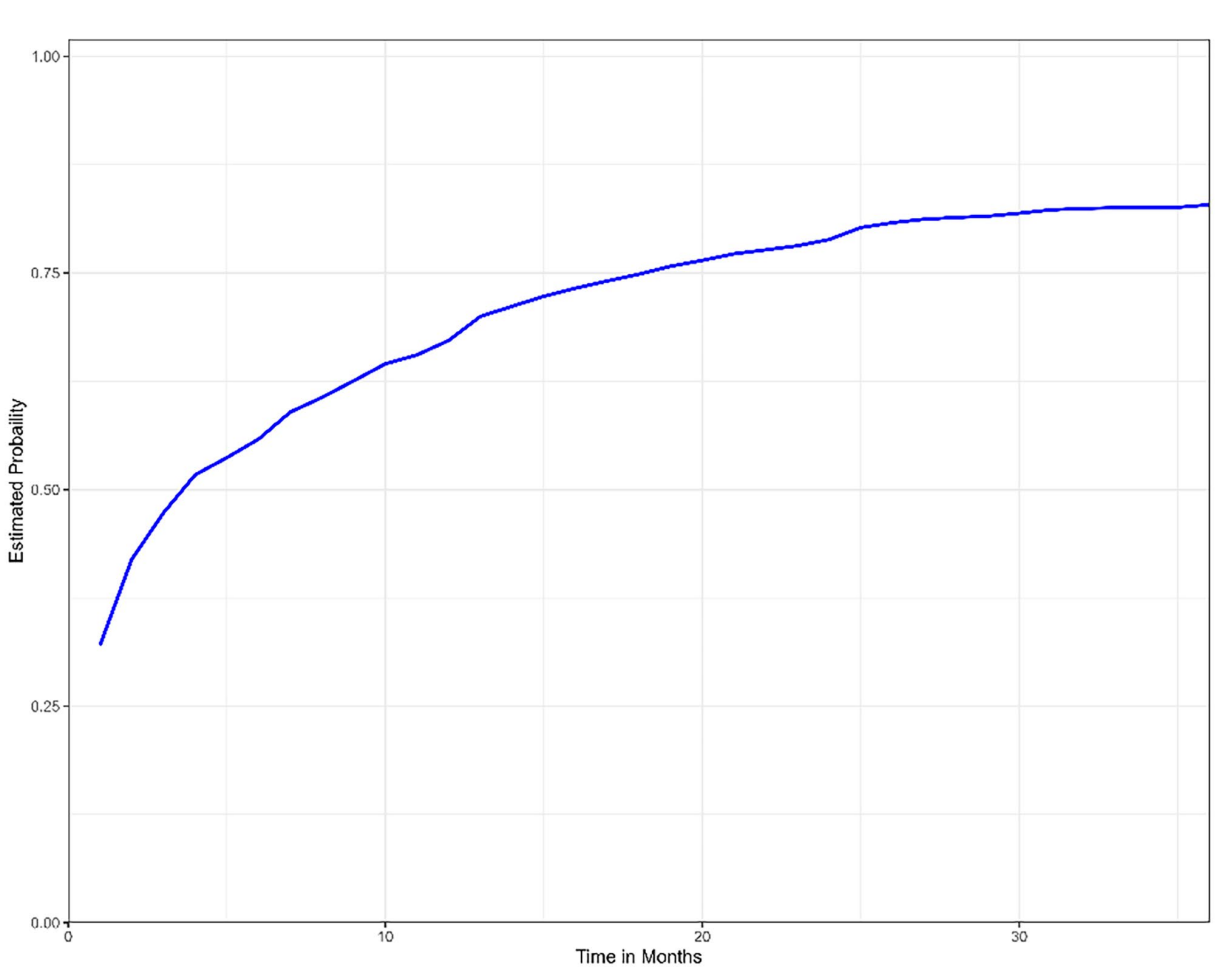
Cumulative probability of contraceptive use at certain time after delivery

One month after delivery, on average, 32% of all women who had childbirth had already started a modern contraceptive method. This increased to 47% after three months and to 55% after six months. Note that these are the cumulative probabilities. One year after delivery, 67% of women started modern contraceptive methods. A total of 79% and 83% of women started modern contraceptives within two and three years after delivery, respectively. Table 3; Fig. 1 show the cumulative probabilities of using postpartum contraception after a certain time.

**Table 3:** Probability for contraceptive use at certain time after deliver

Time (Months)	Contraceptive use Probability
1	0.3201723
2	0.4194185
3	0.4730842
6	0.5582429
12	0.6721959
24	0.7886684
36	0.8288316

## Bivariate Cox model analysis

Initially, 24 variables were selected for bivariate Cox proportional hazard model analysis. Table 4 shows the results of the bivariate analysis.

Bivariate analysis revealed that an increased number of antenatal visits, an increased number of children under five years, women who delivered by cesarean section, women with access to health insurance, and Catholic and Muslim women were associated with an increased usage of contraceptive methods after delivery. In addition, women who had any level of education were associated with a higher use of postpartum family planning compared to women with no education. Compared to women living in Kigali, those living in the North, South, and Eastern provinces had higher odds of using postpartum contraceptives.

On the other hand, an increase in women’s age, number of household members, number of children born, and ideal number of children were associated with reduced chances of using family planning after delivery. Women who delivered at home were significantly associated with a reduction in the use of postpartum family planning compared with women who delivered at health centers. Women who never lived in union, were separated, divorced, or widowed were associated with reduced use of postpartum family planning compared to women who were married and/or living with partners. Lastly, women who reported challenges in obtaining money for healthcare and those who reported difficulty with distance to health facilities were associated with a reduced chance of using postpartum family planning. Similarly, women who lived in households headed by Female were associated with a reduced use of postpartum family planning.

## Multivariable Cox model analysis

Significant and non-negligible variables in the univariate analysis were selected for multivariate analysis. In total, 16 variables were selected for the multivariable analysis. The goodness-of-fit of the model was evaluated using the likelihood ratio test with a *P* value below 0.001. Individual covariates were evaluated based on their coefficients, hazard ratio, and associated *P*-values. Table 5 presents the results of the multivariable Cox Analysis. The test for the Cox proportional hazard assumption was performed using scaled Schoenfeld Residuals.

**Table 4:** Bivariate analysis with Cox proportional hazard model of independent variables on the time of resumption or initiation of contraceptive after delivery

Characteristic	HR^*1*^	95% CI^*1*^	*P* - value
Respondent current age	0.98	0.97–0.98	< 0.001
Total children ever born	0.94	0.93–0.95	< 0.001
Number of antenatal visits	1.09	1.06–1.13	< 0.001
Ideal number children	0.91	0.89–0.93	< 0.001
Timing of 1st antenatal care	0.94	0.92–0.96	< 0.001
Age in 5 - year groups	0.89	0.87–0.91	< 0.001
Number of household members	0.93	0.92–0.95	< 0.001
Number children under5 in household	1.09	1.04–1.14	< 0.001
Antenatal care provider			
Medical doctor	—	—	
No antenatal care	0.69	0.52–0.91	0.008
Nurse/midwife	1.02	0.88–1.19	0.8
Last child delivered by			
Medical doctor	—	—	
Nurse/midwife	0.90	0.84–0.97	0.005
Other	0.52	0.45–0.61	< 0.001
Place of last delivery			
Health center	—	—	
Home	0.53	0.46–0.63	< 0.001
Other	0.75	0.58–0.98	0.033
Private clinic/polyclinic	0.83	0.66–1.05	0.12
Provincial/district hospital	1.06	0.99–1.13	0.12
Referral hospital	1.02	0.89–1.16	0.8
Religion			
Adventist	—	—	
Catholic	1.15	1.04–1.27	0.005
Muslim	1.26	1.00–1.58	0.047
Other	0.89	0.70–1.14	0.4
Protestant	0.99	0.90–1.09	0.8
Current marital status			
Married/Living with partner	—	—	
Never in union	0.54	0.48–0.60	< 0.001
Widowed/Divorced/Separated	0.46	0.41–0.52	< 0.001
Highest Level of education			
No Education	—	—	
Primary	1.38	1.24–1.53	< 0.001
Secondary	1.42	1.26–1.60	< 0.001
Higher	1.32	1.11–1.58	0.002
Wealth Category			
Poorest	—	—	
Poorer	1.11	1.02–1.22	0.020
Middle	1.15	1.05–1.25	0.003
Richer	1.06	0.97–1.17	0.2
Richest	0.97	0.89–1.07	0.6
Type pf Residence			
Rural	—	—	
Urban	0.90	0.84–0.97	0.007
Had terminated pregnancy			
No	—	—	
Yes	0.87	0.80–0.95	0.001
Delivered by caesarean section			
No	—	—	
Yes	1.27	1.17–1.38	< 0.001
Have medical insurance			
No	—	—	
Yes	1.24	1.14–1.34	< 0.001
Problem getting money for medical			
No	—	—	
Yes	0.89	0.84–0.94	< 0.001
Problem distance to health facility			
No	—	—	
Yes	0.89	0.83–0.96	0.002
Respondent currently working			
No	—	—	
Yes	1.02	0.95–1.10	0.6
Province & Region			
Kigali	—	—	
South	1.17	1.05–1.31	0.005
West	0.89	0.79–0.99	0.035
North	1.34	1.19–1.50	< 0.001
East	1.22	1.10–1.36	< 0.001
Sex of household head			
Male	—	—	
Female	0.64	0.59–0.68	< 0.001

^*1*^ HR = Hazard Ratio - CI = Confidence Interval

Multivariable analysis revealed that women who delivered via cesarean section had an increased chance of starting family planning early compared to women who delivered naturally (hazard ratio:1.18, CI:1.07–1.3, *P*-Value < 0.001). Women who delivered by cesarean section had an 18% increased chance of using modern contraceptives after delivery compared with women with natural birth. Compared to women with no health insurance, women who had access to health insurance had 15% increased odds of using modern contraceptives after delivery (hazard ratio:1.15, CI: 1.05–1.26, *P*-Value < 0.01). The increase in the number of under 5-years children in households increased the use of postpartum family planning (hazard ratio:1.17, CI:1.11–1.25, *P*-value < 0.001). An increase of one under 5-year child, increases the odds of utilizing postpartum contraceptives by 17%.

In multivariable analysis, the total number of children born was associated with an increased use of postpartum family planning (hazard ratio:1.04, CI:1.01–1.08, *P*-value < 0.05). This is different from the bivariate analysis, in which the number of children reduced the odds of using postpartum contraceptives. The use of postpartum contraceptives varies across the Provinces and Regions. Compared to women who lived in Kigali, those who lived in the South, East, and Northern provinces had increased use of postpartum family planning. The effect of religion was statistically significant in Catholic Women who were more likely to use contraceptives than in Adventists.

**Table 5:** Multivariable Cox proportional hazard model

*Variable Name*	*Coefficient*	*HR* ^*1*^	*95% CI HR* ^*2*^		*Z*	*P value*
Age in 5-year groups	-0.18498	0.83	0.8	0.87	-8.63	0.00000
Number of antenatal visits during pregnancy	0.04681	1.05	0.99	1.11	1.699	0.08919
Timing of 1st antenatal check months	-0.00896	0.99	0.96	1.02	-0.543	0.58702
Number of household members	-0.03794	0.96	0.94	0.99	-2.717	0.00657
*Current Marital Status (Married as reference)*						
Never in Union	-0.9688	0.38	0.33	0.44	-13.25	0.00000
Divorced, Separated, Widowed	-0.7788	0.46	0.4	0.53	-10.66	0.00000
Had terminated pregnancy	-0.12186	0.89	0.8	0.98	-2.45	0.01415
Delivered by Cesarean delivery	0.16492	1.18	1.07	1.3	3.449	0.00056
Have insurance	0.1382	1.15	1.05	1.26	2.963	0.00304
*Region (reference as Kigali)*						
South	0.16233	1.18	1.02	1.36	2.217	0.02657
West	-0.12637	0.88	0.76	1.02	-1.698	0.08941
North	0.25628	1.29	1.1	1.51	3.191	0.00141
East	0.27882	1.32	1.14	1.53	3.743	0.00018
*Religion (reference as Adventists)*						
Catholic	0.12607	1.13	1.01	1.27	2.194	0.02823
Muslim	0.2052	1.23	0.97	1.56	1.673	0.09416
Others	-0.03406	0.97	0.77	1.22	-0.291	0.77068
Protestant	-0.02338	0.98	0.88	1.09	-0.426	0.66952
*Highest Education Level (reference: No education)*						
Primary	0.14646	1.16	1.02	1.31	2.287	0.02215
Secondary	0.06157	1.06	0.92	1.23	0.810	0.41766
Higher	-0.09602	0.91	0.73	1.13	-0.864	0.38729
Total children ever born	0.04291	1.04	1.01	1.08	2.463	0.01375
Ideal number of children	-0.09978	0.91	0.88	0.93	-7.026	0.00000
Problem getting money for medical	0.02814	1.03	0.95	1.11	0.733	0.46331
Number of children under 5-years	0.16	1.17	1.11	1.25	5.254	0.00000
Problem distance to health facility	-0.11647	0.89	0.82	0.97	-2.703	0.00685

1: Hazard ratio, 2: 95% Confidence Interval for Hazard ratio

Compared to women with no education, those with primary education had increased odds of using contraceptives after delivery. There were no significant differences between women with secondary and higher education and women with no education.

Women’s Age was associated with reduced use of postpartum family planning (hazard ratio:0.83. CI: 0.8–0.87, *P*-Value < 0.001). An increase from one five-year category age to another five-year category of age, was associated with reduction in use of postpartum contraceptives by 17%. There were significant differences in the use of postpartum family planning among women in different marital status categories. Women who had never been in union were associated with reduced odds of utilizing postpartum family planning (hazard ratio:0.38, CI:0.33–0.44, *P*-value < 0.001). Women who were divorced, separated, or widowed also had a reduction in utilizing postpartum family planning (hazard ratio:0.46, CI:0.4–0.53, *P*-value < 0.001).

In addition, women with a history of terminated pregnancies have reduced odds of using postpartum contraceptives. The increase in the number of household members was also associated with reduced odds of utilization of postpartum family planning (hazard ratio:0.96, CI:0.94–0.99, *P*-value < 0.01). An increase in the ideal number of children reported by women was also associated with reduced odds of using family planning after delivery. Similarly, women who reported challenges in accessing health facilities due to long distances were also associated with a reduced use of family planning after delivery.

## Discussion

The use of survival analysis to study family planning after childbirth is a novel approach. Conventional statistical methods such as logistic regression are commonly used for such tasks. However, such methods fail to incorporate follow-up time into the analysis. For instance, in the logistic regression model, the time at which the event of interest, such as the use of family planning after delivery, was not incorporated. Women who used contraceptive methods immediately after delivery and those who used contraceptive at 24th months after delivery received the same weight in logistic regression. However, with the survival analysis strategy, the follow-up times were recorded and incorporated into the analysis. Women who initiated contraceptive use after one month can be distinguished from those who initiated contraceptive use after three or six months without analyzing them independently [16].

This study revealed that 32% of the women used contraceptive one month after delivery. This can be compared to a study conducted at a tertiary hospital in Rwanda, which revealed that 11.4% of the women used immediate postpartum contraceptives. These results are different because the 32% obtained in our study was family planning use after one month, while the study that reported 11.4% only considered immediate postpartum modern family planning. This study revealed that the rate of modern contraceptive use one year after childbirth was 67%. This percentage is comparable to that of a multicenter study conducted in Rwanda, that found one-year postpartum family planning at 72% [9]. Additionally, this percentage was higher than that reported in a study conducted in Ethiopia (56%) [17].

In this study, mode of delivery was the main factor associated with postpartum family planning. Women who delivered via cesarean section had an 18% higher chance of using family planning. An increase in family planning among women who delivered by cesarean section was also reported in a qualitative study conducted in Rwanda that highlighted cesarean section and its associated risks as a motivator for family planning [10]. This result can also be compared with a study in India that found the acceptance of long-term contraceptive use after repeated cesarean sections to be 68%, a much higher rate compared to the general population [18]. The low rate of postpartum contraceptive use in Kigali, compared to the southern, eastern, and northern provinces, was unexpected. Previous reports have indicated higher family planning use in urban areas than in rural areas [19].

This study also observed the effects of religion on postpartum contraceptive use. Compared with Adventists, Catholic women were associated with increased use of contraceptives in both univariate and multivariate analyses. However, there were no statistically significant differences among women of other religions. The relationship between contraceptive behavior and religion has been extensively established. An interesting acceptance of family planning among Catholics, given that the Catholic Church discourages contraceptives use, was also observed in studies conducted in other African countries [20–22]. Lower use of contraceptives among Pentecostal/Protestant women has been reported in studies conducted in Mozambique [21] and Rwanda [10].

Variables related to the number of children per woman also affect modern contraceptive use after childbirth. An increase in the number of children under five years of age was associated with contraceptive use, while an increase in the overall number of children was associated with a reduced chance of using contraceptives after delivery. This is an interesting observation, especially regarding how the overall number of children is associated with a reduced chance of contraceptive use. While no other study has collaborated with the findings, this can be interpreted using socioeconomic factors that are associated with an increased number of children [23].

In addition, this study found that an increase in women’s age was associated with a reduced chance of postpartum contraceptive use. This can be compared to a study that analyzed data from 29 countries and found low contraceptive use among older women [23]. Socioeconomic and related factors were associated with postpartum contraceptive use. Women with no education have a reduced chance of utilizing modern family planning methods. The relationship between education and contraceptive behavior was established in a study that analyzed data from 29 sub-Saharan countries [23].

Finally, this study revealed the impact of marital status on the utilization of postpartum contraceptives. Separated and Divorced women as well as women who had never been in union were associated with reduced contraceptive use after delivery. This can be explained by a study on marital status and family planning that analyzed data from 29 countries. This study found that separated and divorced women were associated with overall low contraceptive use. In contrast, women who had never been married were associated with higher condom use [24].

## Study limitation

This research possesses limitations that should be acknowledged. First and foremost, the study relies on secondary data collected in the 2020 Rwanda Demographic and Health Survey, which may be subject to recall bias. In DHS, participants were asked to recall events related to their reproductive history over a period of up to five years. Such retrospective data may introduce inaccuracies or omissions in reporting, potentially affecting the precision of our findings.

Secondly, while we employed survival analysis techniques, which are suitable for time-to-event data, the study design is cross-sectional in nature, which limits our ability to establish causality between identified factors and the utilization of postpartum family planning methods. Third, this analysis is based solely on the variables available in the DHS dataset, and there may be unmeasured or unobserved confounding factors that influence the outcomes of interest.

Finally, as with any secondary data analysis, the study’s results are contingent on the quality and comprehensiveness of the original data collection process, which may be subject to errors or limitations beyond our control. Despite these constraints, this research provides valuable insights into the factors associated with postpartum family planning use among Rwandan women and can inform future investigations and policy efforts in this critical area of reproductive health.

## Conclusion

The importance of Postpartum Family Planning for the prevention of maternal and child mortality is well established. Understanding the factors that influence the decision to use postpartum contraception is paramount. Factors such as the mode of delivery, female age, number of children born, and ideal number of children influence the decision to use postpartum contraceptives. Socioeconomic factors such as a low level of education, religious affiliation, and marital status were also associated with postpartum family planning use. Policymakers and healthcare providers should provide customized interventions to women in the identified groups to increase the use of postpartum family planning.

## Data Availability

The data utilized in this research are available on DHS Program website on this link: https://dhsprogram.com/data/available-datasets.cfm. The dataset used for analysis after data cleaning and processing is not publicly available due to restrictions of DHS Program prohibiting sharing of data. However, they are available from the corresponding author given reasonable request.
